# Correction: Maldonado Moreno et al. Analgesic and Gastrointestinal Effects of Methadone in Horses Undergoing Orchiectomy. *Animals* 2025, *15*, 2358

**DOI:** 10.3390/ani15223252

**Published:** 2025-11-10

**Authors:** Natalya Maldonado Moreno, Júlia Alves Moreira, Luiza Araujo De Oliveira, Amaranta Sanches Gontijo, Maria Luiza Castilho Baldi, Raphael Rocha Wenceslau, Andressa Batista da Silveira Xavier, Juan Felipe Colmenares Guzmán, Suzane Lilian Beier

**Affiliations:** Department of Clinical and Veterinary Surgery, Veterinary School, Universidade Federal de Minas Gerais, UFMG, Belo Horizonte 31270-901, MG, Brazil; natalyamaldonadomoreno@gmail.com (N.M.M.); juliaa.moreira@yahoo.com.br (J.A.M.); contatoluizaaraujo27@gmail.com (L.A.D.O.); mariacastilhoobh@gmail.com (M.L.C.B.); rwenceslau@hotmail.com (R.R.W.); felipecolmenares94@gmail.com (J.F.C.G.)

**Addition of Authors**

Juan Felipe Colmenares Guzmán and Andressa Batista da Silveira Xavier were not included as authors in the original publication [1]. The corrected Author Contributions statement appears here. The authors state that the scientific conclusions are unaffected. This correction was approved by the Academic Editor. The original publication has also been updated.

**New Author Contributions Statement**

Author Contributions: Conceptualization, N.M.M., A.B.d.S.X. and S.L.B.; data curation, J.A.M., N.M.M., J.F.C.G., S.L.B. and R.R.W.; formal analysis, R.R.W.; funding acquisition, N.M.M. and S.L.B.; investigation, N.M.M., J.F.C.G., and S.L.B.; methodology, N.M.M., J.A.M., A.S.G., L.A.D.O., A.B.d.S.X. and M.L.C.B.; project administration, S.L.B.; resources, S.L.B. and A.B.d.S.X.; supervision, R.R.W., A.B.d.S.X. and S.L.B.; validation, N.M.M. and S.L.B.; writing—original draft, N.M.M.; writing—review and editing, N.M.M., J.A.M., A.S.G. and R.R.W. All authors have read and agreed to the published version of the manuscript.
